# Hepatocellular carcinoma metastasis to the lacrimal gland: A case report

**DOI:** 10.3892/ol.2014.2191

**Published:** 2014-05-28

**Authors:** HUA CHEN, JINPENG LI, LIJUN WANG, NING CONG, CONGCONG SHI, JINLONG SONG, WENZHE BU

**Affiliations:** 1 Department of Surgical Oncology (Interventional Therapy), Shandong Cancer Hospital and Institute, Shandong Academy of Medical Sciences, Jinan, Shandong 250117, P.R. China; 2 Department of Computerized Tomography, Shandong Medical Imaging Research Institute, Jinan, Shandong 250000, P.R. China; 3 Six Ward of Shandong Mental Health Center, Jinan, Shandong 250014, P.R. China

**Keywords:** hepatocellular carcinoma, metastasis, lacrimal gland

## Abstract

Hepatocellular carcinoma (HCC) is a globally common neoplasm, with regional metastasis associated with >50% of the tumors. Extrahepatic metastasis is also common, with the lungs, diaphragm, abdominal lymph nodes and bone being the most frequently affected regions. However, to the best of our knowledge, HCC metastasis to the lacrimal gland has not been reported in the literature. Only one case of metastasis to the lacrimal sac from a renal cell carcinoma has been reported. The current study presents the case of a 56-year-old male with ocular symptoms who was eventually diagnosed with HCC. The therapeutic alternatives for such cases are also discussed according to the reviewed literature. Clinicians should be watchful for the appearance of tumors in the lacrimal gland in patients with a history of malignancy.

## Introduction

The main causes of mortality for patients with hepatocellular carcinoma (HCC) are cancer progression, tumour recurrence and metastasis. Emerging treatment options, such as surgery, interventional radiology, ethanol injection and radiofrequency ablation, have improved the prognosis of HCC (1); however, there are few treatment options for extrahepatic metastases, for which the prognosis is poor. The incidence of extrahepatic metastasis among all HCC patients is reported to be >25%, with the lungs, diaphragm, abdominal lymph nodes and bone recognized as the most frequently affected areas (2). Current treatment options for metastases include surgical excision, radiotherapy, and chemotherapy. Surgical excision may benefit patients with single or regional lesions; however, it is unsuitable for patients with multiple lesions. This report describes a patient for whom a solitary lacrimal gland tumor was the first symptom of HCC. A craniectomy was performed and the mass was totally removed. The histological diagnosis was lacrimal gland metastasis from HCC. The patient was subsequently treated by transcatheter arterial chemoembolization (TACE). To the best of our knowledge, there have been no previoulsy reported cases of lacrimal gland metastases from HCC. Only one case of metastasis to the lacrimal sac from a renal cell carcinoma has been reported (3). The current study presents the first case of this unusual metastatic spread of HCC.

## Case report

**Figure 1:**
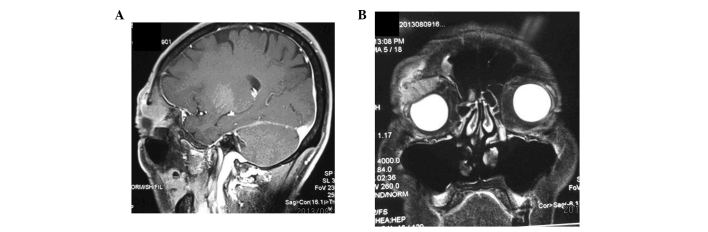
(A) Enhanced magnetic resonance imaging (MRI) of the skull revealing a smooth circular 35×30-mm lesion within the right lacrimal gland, approaching the right frontal plate barrier and the outside eyelids of the right orbit. (B) The cystic signal of the right frontal sinus revealing mild to moderate enhancement in enhanced scan imaging.

**Figure 2:**
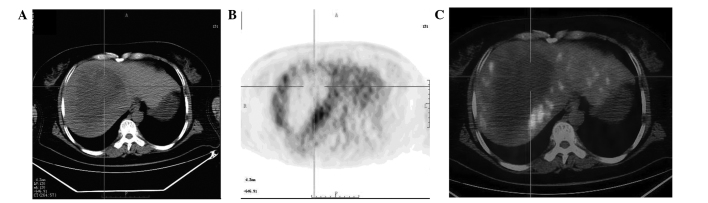
(A) Multiphasic CT images showing a large heterogeneous mass in the right lobe of the liver. (B) PET and (C) PET-CT images revealing an uneven ^18^F-fluorodeoxyglucose uptake shadow in the marginal portion of tumor, particularly within the upper edge of the tumor. The SUV_max_ was 10.3, while with the delayed scan the SUV_max_ was 13.2. CT, computed tomography; PET, positron emission tomography, SUV_max_, maximum standard uptake volume.

**Figure 3:**
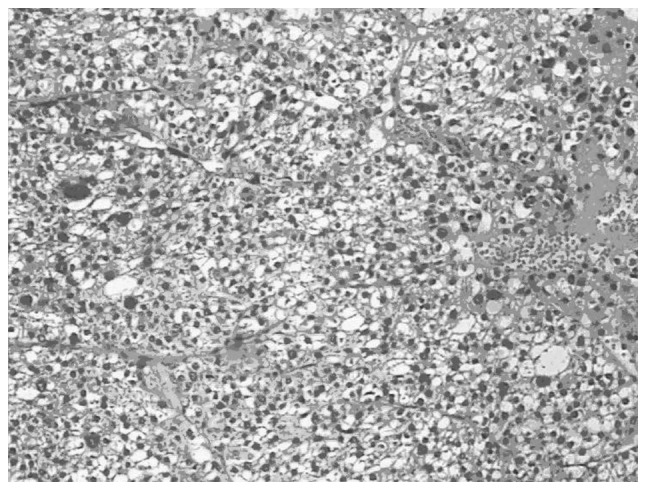
High-power view of a poorly-differentiated adenocarcinoma. Immunhistochemistry was consistent with a hepatic origin (magnification, ×400).

A 56-year-old male was admitted to Shandong Cancer Hospital and Institute (Jinan, China) due to a 15-day history of epiphora associated with a non-tender 35×30-mm pulsatile mass in the right lacrimal gland and the loss of 3 kg of weight in one month. Upon physical examination, the patient was conscious with normal vision, but exhibited optic nerve palsies and hepatosplenomegaly, with no signs of ascites. The liver was palpable ~5 cm below the right costal margin, with a hard and irregular edge. The spleen was marginally enlarged. The superficial lymph nodes were not enlarged. Furthermore, the complete blood count, clotting profile, blood glucose level and liver and renal function tests were normal. However, the serum α-fetoprotein level was elevated to 2,420 ng/ml, and the hepatitis B virus surface antigen status was positive. Chest X-ray results were normal, however, the abdominal ultrasonography revealed a solid 7.5×5.5-cm mass in the right hepatic lobe, while the remainder of the liver was normal. Computed tomography (CT) images revealed a smooth circular lesion in the right orbit and a mucosal cyst in the left maxillary sinus, which required further examination. In addition, magnetic resonance imaging (MRI) scans revealed a smooth circular lesion within the right lacrimal gland and right frontal plate barrier, indicating the possibility of a metastatic tumor or primary lacrimal migraine invasion (Fig. 1). ^18^F-fluorodeoxyglucose positron emission tomography/CT revealed high uptake in the right lacrimal gland and right orbital bone, and heterogeneous uptake in the right lobe, indicating a puncture (Fig. 2). The tumor was excised and the pathological analysis showed a poorly-differentiated clear cell carcinoma. A histological review of the resected specimen revealed a metastatic HCC (Fig. 3), with broad-spectrum immunohistochemical staining for CK (+), GPC-3 (+), HMB45 (−), CK7 (−) and CK8/18 (−).

The carcinoma was radically resected with the surrounding normal tissue under general anesthesia. Histopathological examination of the carcinoma revealed pleomorphic tumor cells with eosinophilic cytoplasms and prominent nucleoli and mitosis arranged in trabecular and solid pattern which confirmed the diagnosis of metastasic HCC. Following seven days rest, the patient was treated with TACE, consisting of 30 mg epirubicin, 50 mg cisplatin and 10 ml iodized oil and clinically monitored for disease progression. Recent studies have demonstrated that TACE effectively controls the symptoms of advanced HCC, even in patietns with vascular invasion or metastases (4,5). Due to the fact that there was only a single lacrimal tumor, the patient did not undergo radiotherapy. Postoperative recovery was satisfactory. The patient was discharged one month later. After six months of follow-up the patient did not exhibit any evidence of recurrence in the lacrimal gland. However, the patient exhibited recurrence of the HCCs and succumbed to liver failure in the 12 months after receiving TACE.

## Discussion

HCC exhibits a widely varying incidence in different countries, however, its worldwide prevalence makes it one of the most common malignancies. HCC occurs most frequently in elderly males with alcoholic liver cirrhosis. In total, >50% of HCC cases result in extrahepatic metastases (6,7), which occur most frequently in the lungs, adrenal glands and regional lymph nodes (8,9), but rarely in the lacrimal gland. Metastases to the lacrimal gland are extremely rare, however, when they do occur, they appear as exophytic tumors, and rarely with bone changes. In the current study, the patient presented with a non-tender pulsatile mass in the right lacrimal gland, and was experiencing pain and an eye movement disorder. Besides the non-typical clinical presentation, the MRI appearance of the lacrimal gland may also aid in the differential diagnosis between cystic carcinoma and metastases, however, the final diagnosis relies on the histopathology or cytology. In the present case, the patient was diagnosed with a metastatic HCC tumor of the lacrimal gland, which was confirmed by biopsy. Thus far, no studies have investigated therapeutic approaches for the treatment of lacrimal gland metastatic disease. The current patient was treated with TACE and clinically monitored for disease progression. Current treatment options for lacrimal gland metastases of HCC or non-HCC include surgical excision, radiotherapy and chemotherapy. Surgical excision may benefit patients with single or regional lesions. However, for HCC patients with >2 metastatic lesions surgery and radiotherapy are unsuitable due to excessive radiation damage to the surrounding tissue (10). Chemotherapy is suitable for patients with multiple lesions, but it is not particularly sensitive and has a poor prognosis (11,12).

In conclusion, the current study presents a case of unusual HCC metastasis in the lacrimal gland. The clinical diagnosis was difficult, however, metastasis must always be considered in patients with a history of malignancy. Laboratory and imaging studies do not aid the diagnosis and therefore, only fine-needle aspiration and/or biopsy may confirm it. The therapeutic options for this condition have not been well-studied and the effects require further follow-up.
